# Usability and feasibility of a cognitive-behavioral mobile app for ADHD in adults

**DOI:** 10.1371/journal.pdig.0000083

**Published:** 2022-08-15

**Authors:** Laura E. Knouse, Xiaodi Hu, George Sachs, Sebastian Isaacs

**Affiliations:** 1 Department of Psychology, University of Richmond, Richmond, Virginia, United States of America; 2 Master of Science in Human-Computer Interaction Program, College of Information Studies, University of Maryland, College Park, Maryland, United States of America; 3 Get Inflow, LTD, London, United Kingdom; University of Bayreuth: Universitat Bayreuth, GERMANY

## Abstract

**Objective:**

Cognitive-behavioral therapy (CBT) has growing evidence of efficacy for Attention-Deficit/Hyperactivity Disorder (ADHD) in adults. Mobile health apps are promising tools for delivering scalable CBT. In a 7-week open study of Inflow, a CBT-based mobile app, we assessed usability and feasibility to prepare for a randomized controlled trial (RCT).

**Method:**

240 adults recruited online completed baseline and usability assessments at 2 (*n* = 114), 4 (*n* = 97) and after 7 weeks (*n* = 95) of Inflow use. 93 participants self-reported ADHD symptoms and impairment at baseline and 7 weeks.

**Results:**

Participants rated Inflow’s usability favorably, used the app a median of 3.86 times per week, and a majority of those using the app for 7 weeks self-reported decreases in ADHD symptoms and impairment.

**Conclusion:**

Inflow demonstrated usability and feasibility among users. An RCT will determine whether Inflow is associated with improvement among more rigorously assessed users and beyond non-specific factors.

## Author summary

Attention-Deficit Hyperactivity Disorder (ADHD) is characterized by frequent and impairing inattention and/or hyperactivity/impulsivity that begins in childhood and sometimes continues to cause problems into adulthood. While some medications are helpful in treating ADHD, medications do not work for everyone and some adults continue to experience ADHD symptoms even with medication treatment. Cognitive-behavioral therapy (CBT) helps people learn skills to better manage their actions and thoughts. Face-to-face CBT for adult ADHD has shown promising results in some studies, but this treatment can be hard for people to access. Mobile apps are promising tools for delivering CBT to more people in their daily lives but only one previous study has examined a CBT-based app for adults with ADHD. To prepare for a larger controlled study, we gathered data from participants who used Inflow, a CBT-based mobile app, for 7 weeks. We recruited 240 adults online, gave them access to the app, and asked those who downloaded it (*n* = 205) to complete assessments after 2, 4, and 7 weeks. Participants rated Inflow as user-friendly and we learned more about how often people use they app and which features they access. A majority of the people who stayed in the study after 7 weeks (*n* = 93) rated their ADHD symptoms as less severe than when they started the study. Next, we need to conduct a randomized, controlled study to better evaluate whether these changes are really due to the effects of Inflow.

## Usability and feasibility of a cognitive-behavioral mobile app for ADHD in adults

Attention-Deficit/Hyperactivity Disorder (ADHD) is a neurodevelopmental disorder characterized by frequent and impairing inattention and/or hyperactivity/impulsivity that begins in childhood but continues to cause problems into adulthood for about 2/3 of people diagnosed [1]. ADHD in adults is associated with functional impairment in a variety of domains including education, work, interpersonal relationships, and physical and mental health and well-being [2]. With an estimated prevalence of 4.4% in the U.S. population [3], ADHD in adulthood is associated with substantial individual and societal economic costs [4] and even reduced estimated life expectancy [5]. Stimulant medications have demonstrated efficacy in the treatment of adult ADHD [6,7]. However, these medications do not work for every adult with ADHD and even adults who experience a positive response to these medications may continue to experience clinically significant symptoms and impairment in need of intervention [8].

The most well-studied non-medication treatment approach for adult ADHD is cognitive behavioral therapy (CBT), which targets aspects of thoughts and behaviors that contribute to ADHD-related symptoms and impairment. CBT helps clients to develop and implement self-regulation skills and to manage thoughts that may impede their behavior change [9,10]. Importantly, to gain and sustain new skills, patients need to practice skills outside of CBT sessions and implement them in daily life [11,12]. Results from CBT-based treatments for adult ADHD have been promising in prior open trials and randomized controlled trials (RCTs), although available data remain somewhat limited [13–15]. For example, Safren and colleagues [16] found that a 12-session individual CBT program for adults with ADHD who had been treated with medication but who still experienced symptoms resulted in superior symptom reduction compared to a control treatment (relaxation training).

Despite its potential efficacy, traditional CBT for adult ADHD has some disadvantages. First, traditional individual and group CBT has **limited accessibility**. These treatments often have a high barrier to entry, as they require clients to meet in person on a regular basis with a therapist, often in an on-site clinic, which can be inconvenient for people with certain disabilities or comorbid disorders [17]. Furthermore, access to therapists who are well trained in CBT for adult ADHD is limited and may not be available outside of urban centers. Second, traditional CBT for adult ADHD may have a **high cost** for clients, particularly in the United States healthcare system [18]. Finally, the skills taught in traditional CBT for ADHD can be **challenging to implement in daily life**, since treatment often takes place in an entirely different context (i.e., the clinic) from the settings in which skills will need to be implemented. For adults with ADHD, completing skills practice outside of sessions has been associated with greater benefit from CBT [19], yet following through with skills practice in daily life can be especially challenging for people with ADHD, given the executive functioning deficits associated with the disorder [20].

The increasing prevalence of mobile phone usage has paved the way for mobile health applications (mHealth apps), which may address some of the limitations of traditional therapy [21]. Some apps work in tandem with traditional CBT to better track patient’s symptoms and use of skills outside of therapy sessions: for example, CBT-I Coach enhances traditional CBT for insomnia [22,23]. Other apps are designed to be used independent of traditional CBT. Examples of this type include PRIME, an mHealth app designed to improve motivation and quality of life for young people with schizophrenia [24] and Zemedy, an mHealth app designed to increase access to CBT-based treatment for irritable bowel syndrome at scale [25]. Importantly, although data on the efficacy of self-contained CBT-based mHealth apps are currently limited, some promising findings are emerging. For example, in a randomized crossover trial, use of Zemedy was associated with significant reductions in IBS-related symptoms and depressive symptoms as well as improvements in quality of life [25].

A few studies have investigated the efficacy of internet-based CBT interventions for adult ADHD with promising results [26,27]. Recently, Jang and colleagues [28] reported positive usability and feasibility results for a chatbot-based app designed to provide psychoeducation and CBT self-help skills to adults with attention problems. In a small randomized controlled trial (*n* = 46) they found that, over four weeks, app users showed greater reductions in hyperactive-impulsive and overall ADHD symptoms than a group given an ADHD self-help book to read. This study suggests that apps promoting CBT-based ADHD psychoeducation and skills-based treatment may be a promising approach; however, to our knowledge, no other peer reviewed studies have investigated a CBT-based mHealth app specifically designed for adult ADHD. This represents a significant gap in the literature. A systematic review of available apps for ADHD identified 109 apps, 33 of which were designed for adults, but none of these reported or were supported by evidence of efficacy [29]. There is clearly a need for rigorous, systematic evaluations of app-based interventions for ADHD.

The primary aim of the current study was to assess the usability and feasibility of Inflow, a novel mHealth app designed to deliver CBT for adult ADHD, toward the design of a randomized controlled trial. Importantly, Inflow is not a productivity tool such as a calendar or task list app; rather, it provides CBT-based psychoeducation and guides users in implementing new ADHD-relevant behavioral and cognitive skills in daily life. Inflow also allows clients to track their progress and access a supportive community of other users who are engaged with the app.

We conducted a seven-week open feasibility study of Inflow. The primary aims of this preregistered study were to assess the usability and feasibility of the app for users. We hypothesized that the mean score on a usability scale at 2, 4, and 7 weeks would be greater than 3 (neither agree nor disagree) to a statistically significant degree, indicating positive experiences with app usability. We assessed feasibility via descriptive analysis of various aspects of users’ interactions with Inflow. We also collected participants’ self-reported data on ADHD symptoms and functioning to inform the design of a future RCT [30].

## Method

### Participants

Study data are available on Open Science Framework at https://osf.io/qa26m/. Adult volunteers from the United States (*n* = 241) were recruited from Inflow’s interest waitlist. Participants were required to be 18 years or older and were not required to report a prior ADHD diagnosis. Target sample size was determined based upon expected effect sizes on the exploratory efficacy measures and anticipated dropout rate. See Table 1 and Participant Characteristics section in Results for participant demographics.

**Table 1:** Description of Baseline and Week 7 sample.

	Baseline Sample (*n* = 240)	Week 7 Sample (*n* = 93)
	*M* (*SD*), Min-Max	
Age	29.15 (7.14), 18–46	31.49 (7.14), 18–45
	Percentage	
Gender		
Man	21.3	19.4
Woman	69.2	71.0
Other (e.g., non-binary)	9.6	9.7
Race*		
While/Caucasian	78.8	79.6
Black/African American	9.2	10.8
Native American or American Indian	4.2	3.2
Asian/Pacific Islander	5.8	4.3
Other	9.6	9.7
Hispanic/Latinx		
Yes	17.1	17.2
No	82.9	82.8
Education Level		
No high school diploma	2.9	2.2
High school diploma	3.3	4.3
Some college (includes current students)	32.1	26.9
Associates or Technical degree	6.7	4.3
Bachelor’s degree	35.8	36.6
Master’s degree	15	19.4
Doctoral, Medical, or Law degree	4.2	6.5
Marital Status		
Single	47.1	45.2
Married	24.2	33.3
Living with partner	24.2	17.2
Divorced	4.6	4.3
Widowed	0	0
Has children		
Yes	22.9	28.0
No	77.1	72.0
Ever diagnosed with ADHD		
Yes	85.8	88.2
No	14.2	11.8
Currently diagnosed with ADHD		
Yes	84.2	87.1
No	15.8	12.9
Currently taking meds for ADHD		
Yes	60.4	61.3
No	39.6	38.7
Current receiving non-med treatment for ADHD		
Yes	22.9	26.9
No	77.1	73.1
Self-Reported Current Diagnoses		
Any mood disorder	42.9	40.9
Depressive Disorder	40.4	37.6
Bipolar Disorder	3.3	4.3
Other Mood Disorder	0.4	1.1
Any anxiety disorder	53.8	48.4
PTSD	13.3	14.0
Generalized anxiety disorder	9.6	7.5
Social anxiety disorder	3.8	4.3
Specific phobia	0.4	0.00
OCD	4.2	3.2
Panic disorder	1.7	1.1
Agoraphobia	0.4	1.1
Other Anxiety Disorder	35.4	32.3

Note

*Percentages do not sum to 100 because participants could check all that apply for this variable. ADHD = Attention-Deficit/Hyperactivity Disorder. PTSD = Post-Traumatic Stress Disorder. OCD = Obsessive-Compulsive Disorder.

### Development and description of inflow app

Inflow is a science-based app to help people manage their ADHD. Inflow is designed to be a self-help program, based on the principles of CBT for ADHD. It has been developed by a team of clinicians and technologists, including clinicians with expertise in the ADHD CBT field. Throughout the development of Inflow, developers continuously assessed usability by tracking analytics events to monitor user interaction and to better understand what percentage of users were able to successfully perform certain actions. Developers also conducted contextual interviews and usability testing via video call to better understand any usability issues. Data from these sources were used internally to make improvements and refinements to the user experience.

**Fig 1:**
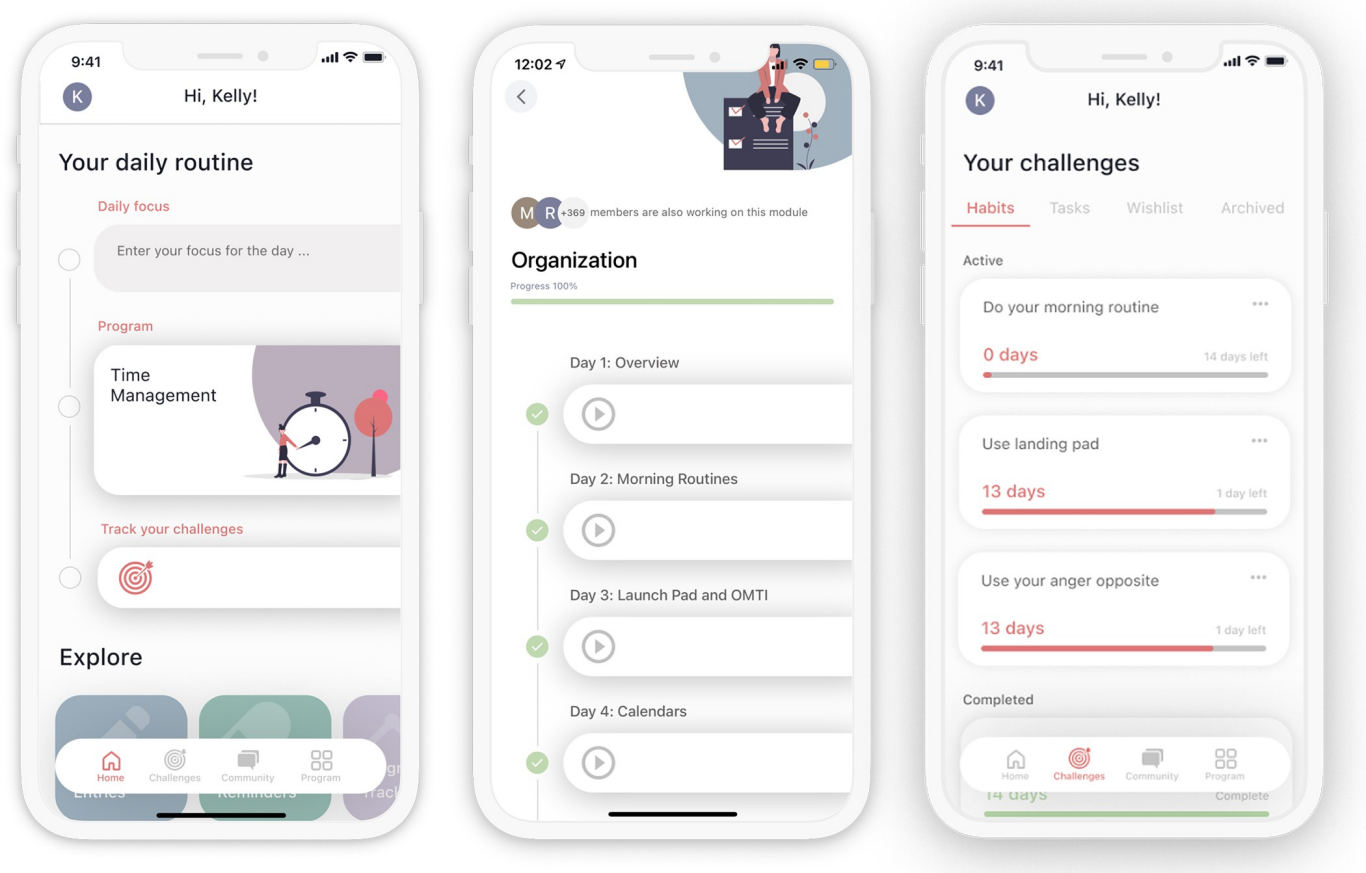
**Screenshots of homescreen (a), program module with exercises (b), and tracked challenges (c).** Panels are ordered a, b, and c left to right.

Users are onboarded onto the app and asked to complete a few questions such as their age and their goals for improvement. Users are then taken to the home screen, which is the main navigation point where they are directed to the tasks and tools they should utilize on a daily basis. On the home screen (Fig 1A) users can see their Daily Routine and tools. The daily routine is a list of tasks for the user on that day. The first item is the Daily Focus where the user is prompted to set a goal by inputting their personal main objective for the day. The second item on the daily routine is the program, which provides core CBT psychoeducation. When a user first joins the app they are offered an introductory module which provides them with an overview of ADHD, CBT, and the usage of the app. On the program page (Fig 1B) the user sees the **modules** that make up the CBT program. Each module focuses on a different area of executive functioning and ADHD management (e.g. Time Management, Organization, Impulsivity). Each module is broken down into daily **exercises**, which includes audio and/or text lessons that provide insight into the specific topic as well as information on strategies to help users manage their symptoms more effectively. The psychoeducational exercises culminate in optional 1- or 14-day **challenges** that track and encourage daily practice of the skills and cement new habits. Inflow members return to the app each day to indicate if they have completed the chosen challenge. An example of a challenge might be to "take three deep breaths when anxious." (Fig 1C).

**Fig 2:**
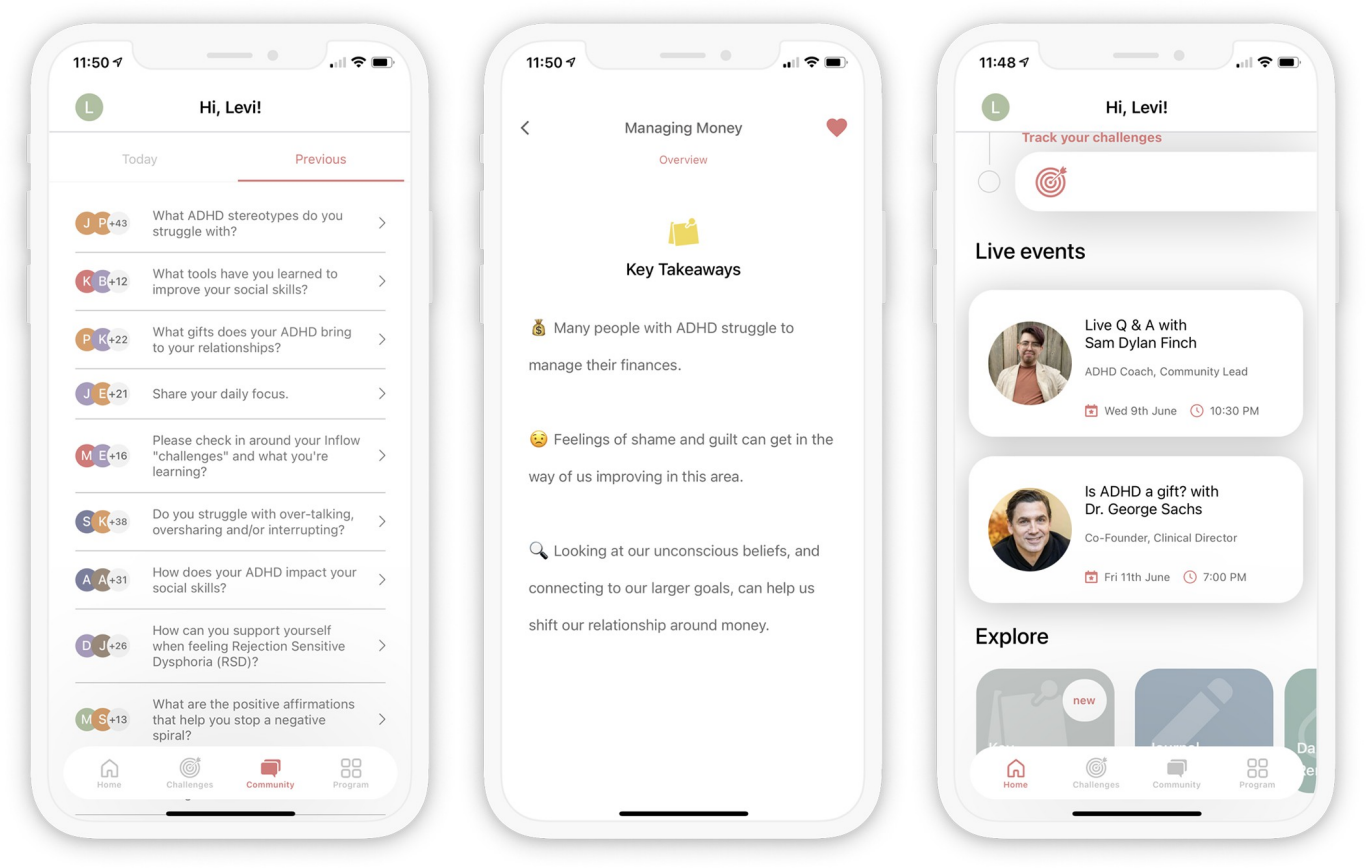
**Screenshots of community posts (a), key takeaways (b), and live events (c).** Panels are ordered a, b, and c left to right.

Inflow members are also given access to a **community** of other app users. This feature aims to provide a sense of social connectedness and social support with other people who are facing similar issues in managing their ADHD and to allow users to share strategies for success. Each day, users are asked a daily question (e.g. How are you feeling today?). They can engage by reading others’ posts in response to the questions, ‘liking’ others’ posts, and posting their own responses (Fig 2A).

Finally, in the Explore section of the home screen there are four additional tools available to users: Daily Reminders, Journal Entries, Key Takeaways, and Live Events. The **journal entries** can also be accessed through the modules and allow users to further analyze their thoughts and behaviors to be reviewed anytime. The Key Takeaways section allows users to save their favorite takeaways from exercises of previous modules so they can be easily reviewed (Fig 2B). The Live Events section allows users to watch current and past in-app **live events** which include weekly live Q&A or guided sessions with ADHD psychiatrists, therapists, and coaches (Fig 2C). Daily reminders enable users to set customized reminders for when to complete different aspects of Inflow—for example, their challenges.

### Measures

#### Usability scale

Usability measures user satisfaction with Inflow and its acceptability. To measure usability, we adapted a scale used by Biagianti et al. [31]. The scale contains 13 items that assess user evaluation of inflow on a 5-point Likert scale (1 = Strongly Disagree; 5 = Strongly Agree). Internal consistency for the usability scale was excellent at 2, 4, and 7 weeks (ɑ = .90, .91, .92, respectively).

#### Feasibility metrics

Feasibility measures users’ engagement with Inflow. To measure feasibility, we used the data collected from the app to analyze frequency and duration of app use, specifically the total number of app sessions during the seven weeks and the median app session duration per participant. We also measured the frequency of specific ways of engaging with the Inflow app, described above, including: completing exercises, tracking and completing challenges, posting in the community and viewing posts, making journal entries, and attending live events hosted by Inflow. We also calculated an *active use rate* which was the sum of the frequency of each of these activities divided by the weeks of use (7) as reported in prior studies [24]. Finally, we measured the number of modules started and completed over the seven weeks.

#### Barkley Adult ADHD Rating Scale (BAARS) [32]

Items from the BAARS were used to measure participants’ ADHD symptoms at baseline and during 7 weeks of Inflow use. We administered the 18 *DSM-IV* items as well as accompanying questions about age of onset and impairment associated with symptoms. Participants responded to the symptom items on a four-point Likert scale (1 = Never or rarely; 4 = Very often). At baseline, they were asked to rate their symptoms over the past six months, per scale instructions, and at seven weeks they were asked to rate symptoms over the prior seven weeks. The scale demonstrates good reliability and validity in past studies [32] and internal consistency was acceptable to good for each subscale—Inattentive, Hyperactive-Impulsive, and Total symptoms—used in our analyses at each timepoint (ɑ = .74-.92). We used the mean scores for each of these subscales in our analyses.

#### Barkley Functional Impairment Rating Scale (BFIS) [33]

The Barkley Functional Impairment Scale (BFIS) was used to measure participants’ self-reported impairment at baseline and during the 7 weeks of Inflow use. The scale asks participants to rate how much they have had difficulty functioning in 15 different major areas of life activity. Participants responded on a 10-point Likert scale (0 = not at all impaired; 9 = severely impaired) or could indicate that a particular area did not apply to them (e.g., parenting) and that area would not be included in the participant’s mean score. At baseline, they were asked to rate their symptoms over the past six months, per scale instructions, and at seven weeks they were asked to rate symptoms over the prior seven weeks. The scale demonstrates good reliability and validity in past studies [33] and internal consistency was good at both time points (ɑ = .86 at baseline; .89 at 7 weeks). We used the mean scores for this scale in our analyses.

### Procedure

**Fig 3:**
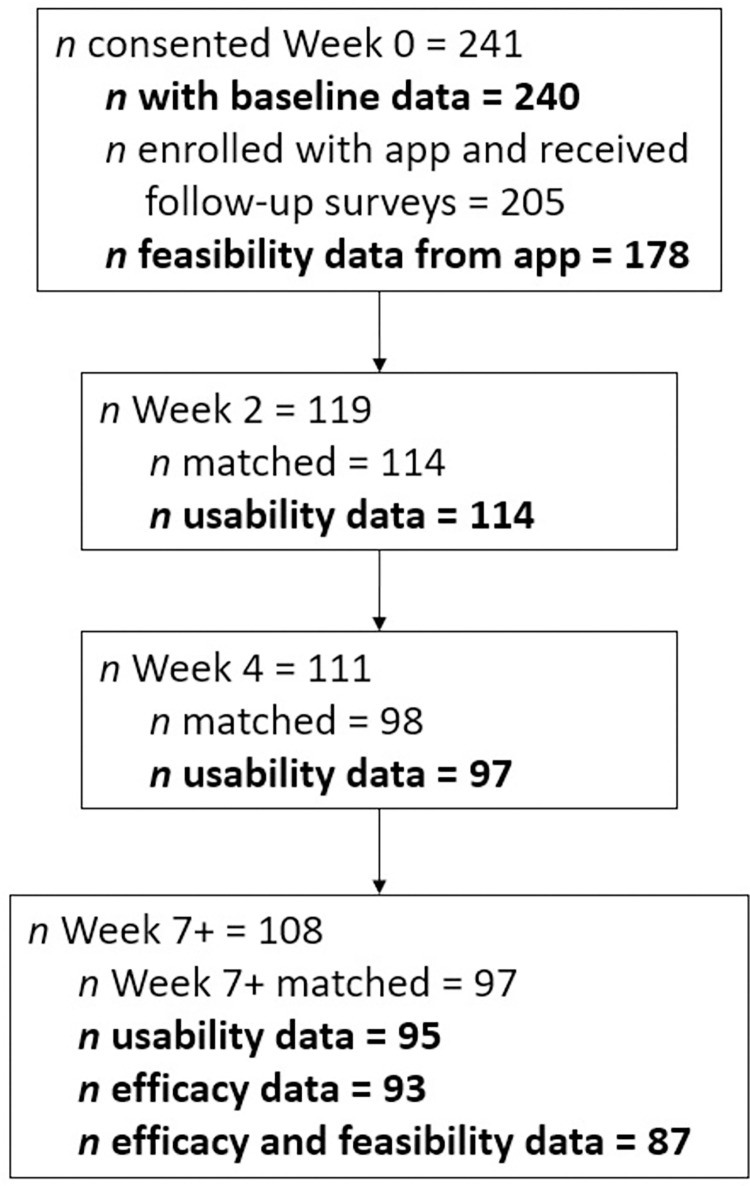
Participant flow and sample sizes at each stage of the study. The label Week 7+ indicates that participants could have completed the final survey more than 7 weeks after baseline. See procedures section for more details.

Study procedures were approved by the Institutional Review Board at the University of Richmond and ethical standards of the American Psychological Association were followed during the conduct of the study. Study procedures and plan of analysis were pre-registered on Open Science Framework at https://osf.io/6zuqc. See Fig 3 for participant flow through the study and *n* at each stage.

#### Recruitment

Participants were recruited from a waitlist of potential users who were interested in gaining access to the Inflow app. Inflow created a 1-page website where people could input their email address to be placed on the interest list. This website was then shared with relevant pages and groups on Facebook. Additionally, a Twitter account was set up to discuss and share the upcoming app. There was considerable interest, with over 10,000 sign-ups over a 6-month period. Consequently, only a small proportion were invited to the join the study. These participants were invited at random. Potential participants from the waitlist received an email describing the research opportunity including a unique registration code and a discount code for the Inflow app and a link to the baseline survey. Participants were instructed to download the app and input the discount code for free access during the 7 weeks of the study and to complete the baseline survey in order to enroll. Participants who did not complete all three steps were sent a reminder email; however, if they did not complete these enrollment steps, they did not receive further surveys. Participants were instructed that, if they completed the assessments, they would receive an additional free year of Inflow

#### Online surveys

Participants completed the baseline survey, starting with the study consent form. They then entered their unique registration code so that their baseline survey data could be linked back to their email address and data generated by their use of the Inflow app, yet this identifying information would stay separate from the survey data.

Next, users were prompted to generate a new unique identifier code by entering the last two digits of their current telephone number, the first and last letter of their middle name (or XX if none), the first letter of the city they were born in (or their hometown city; see Data Screening and Matching section), and the last two digits of their current ZIP code. This unique identifier was used to link participant data across surveys. Next, participants completed demographic questions and gave baseline ratings using the BAARS and BFIS.

Participants who completed full study enrollment (*n* = 205) received emails after 2, 4, and 7 weeks containing links to corresponding surveys. Initial recruitment emails were sent on March 24, 2021, Week 2 survey email was sent on April 7, 2021, the Week 4 email was sent on April 21, 2021, and the Week 7 email was sent on May 12, 2021. Reminder emails for survey completion were also sent at each time point a few days after the initial email. Participants first generated their identifier code using the prompts above and then completed the usability questions. At 7 weeks, they also completed the BAARS and BFIS questionnaires to rate their symptoms and functioning during the previous 7 weeks. Participant responses to the Week 7 survey were downloaded on June 25, 2021 and so Week 7 responses completed before this data were included in the analyses.

#### Data screening and matching

If duplicate surveys were completed by the same participant for the same assessment time point but only one survey provided complete data, the survey with complete data was retained. If more than one set of complete survey data was submitted by a user for a time point within the same 24-hour period, the latest survey was retained. (Often, a user would submit a survey immediately after a prior survey with minor modifications, possibly indicating that they meant to correct their immediately prior response.) However, if two complete surveys were submitted by a user more than 24 hours apart, the first survey was retained. Total *n* in Fig 3 does not include duplicate surveys.

To match the survey data across timepoints, we first removed test and empty survey responses, then located surveys with unique identifiers or IP addresses that could be identically matched to a survey at baseline. Due to experimenter error, one of the questions used to generate participant unique identifiers differed slightly between the baseline and Week 2 surveys vs. the Week 4 and Week 7 surveys. Specifically, for the former, participants were asked for the first letter of the city they were born in and for the latter they were asked for the first letter of the city of their hometown. Thus, for unmatched cases at 2, 4, and 7 weeks, we accepted as valid matches those identifiers for which three out of four of the components matched a survey at baseline and for which location data indicated a difference of less than 1 degree of longitude and latitude. Notably, we compared our results using this matching method with more stringent methods (e.g., requiring either fully ID or IP matches) and results remained unchanged.

### Departures from Pre-Registration

In the pre-registration, our target sample size was 200 participants for the baseline survey; however, we consented 241 participants, 205 of whom successfully enrolled with the app, because we were not able to tightly control how many users responded to the email invitations, which were sent in batches. As detailed in the Procedure, we added methods to handle duplicate surveys and matching procedures in cases where user-generated IDs failed to match across surveys. In addition, we verified that participants had downloaded and enrolled with the Inflow app before sending additional surveys, which we had not specified in the pre-registration. Finally, we modified the way that the feasibility measure of active use rate (see Measures section) was calculated because we realized that our original definition did not incorporate the primary ways that users could have engaged with the app.

### Statistical analyses

Analyses were conducted using IBM SPSS Statistics for Windows, version 28 [34].We first calculated descriptive statistics for participant characteristics (Table 1) and then compared study completers to non-completers using *t*-tests for continuous outcomes and *X*^*2*^ tests for categorical outcomes. For usability analysis, per our preregistered analyses, we used one-sample t tests to determine whether usability ratings were significantly different from the scale midpoint of 3 (neutral). Next, we calculated descriptive statistics (*M*, *Median*, *SD*, *Min and Max*) for app feasibility metrics (app usage data). For exploratory analyses of efficacy, we first calculated change scores for each participant for self-reported ADHD symptoms and impairment by subtracting pre-test scores from post-test scores such that improvement is indicated by a negative score (i.e., a decrease in symptoms and impairment). Next, we calculated pre-to-post effect sizes (*d*) using the *SD* of the baseline score as the standardizer [35] and conducted paired-samples t tests for each self-reported symptom measure and self-reported impairment. Finally, we calculated correlation coefficients (Pearson *r*, 2-tailed tests) between symptom and impairment change scores and the app usage data. Because several app usage variables were significantly skewed, we also calculated and reported these correlation coefficients after removing extreme outliers defined as values more than 3 times the interquartile range from the end of the box in a boxplot [36] for each usage variable. No control variables were used in any analyses.

## Results

### Participant characteristics

See Table 1 for full participant demographics. 241 participants consented and 205 successfully enrolled with the app. Of these, 108 participants completed the Week 7 survey for a retention rate of 52.7% of fully enrolled participants. As illustrated in Fig 3, 178 of consented participants had retrievable feasibility data from the app, 114 participants completed the usability survey at Week 2 and could be matched with their baseline data, 97 matched participants completed the survey at Week 4, and 95 completed the usability survey at Week 7. After Week 7, 93 matched participants provided data for efficacy analyses and 87 of these also had feasibility data from the app. Table 1 also reports the demographic characteristics of the 93 participants who provided efficacy data.

The baseline sample consisted of young to middle aged adults (18–46 years) and the majority identified as women. The majority were white/Caucasian and had attended some college or obtained a Bachelor’s degree. In the baseline sample, 85.8% of participants reported having been diagnosed at some point in their lifetime with ADHD and 84.2% endorsed a current diagnosis. 71.8% of those endorsing a current diagnosis reported currently taking medication for ADHD (60.4% of the overall sample) and 27.2% reported current non-medication treatment for ADHD (22.9% of the entire sample). As is the case in many studies of ADHD in adults (Miller et al., 2007) comorbidity was common with 53.8% of the baseline sample reporting at least one anxiety disorder diagnosis and 42.9% reporting at least one mood disorder diagnosis.

Among the participants who provided data for the efficacy analyses (right side of Table 1), the mean score for ADHD symptom at baseline fell at the 98th percentile based on norms for adults aged 18–39 [32] and the mean impairment score fell between the 92nd and 93rd percentile [33]. Thus, as a group, participants reported clinically significant symptoms and impairment at baseline.

Comparing participants in the baseline sample who provided matchable data at Week 7 (*n* = 97) to those who did not allows for consideration of whether completion of the study was dependent on any baseline characteristics. (Although note that the 97 does not include participants who we were unable to match to their baseline data.) Participants who completed the study tended to be older by, on average, about four years (*M* = 31.48, *SD* = 7.04 vs. *M* = 27.57, *SD* = 6.78), *p* < .001), and were more likely to be married (17.5% of non-completers; 34% of completers; *p* = .02) vs. living with a long-term partner. There was also a trend toward study completers being more likely to have children (18.9% of non-completers, 28.9% of completers; *p* = .07). Notably, study completion did not depend on baseline self-reported ADHD symptoms or impairment, anxiety disorder diagnosis, mood disorder diagnosis, race, ethnicity, gender or level of education (all *p* > .05).

### Usability

At all three timepoints, participants on average somewhat agreed to strongly agreed that Inflow was user-friendly, enjoyable, helpful, and that they would recommend it to others (Table 2). Mean scores at all timepoints were significantly different from the scale midpoint of 3 (Neither Agree nor Disagree), meaning that, on average, participants agreed with all usability statements.

**Table 2:** Mean Scores for Usability Questions at 2, 4, and 7 Weeks.

	Week 2 (*n* = 114) *M* (*SD*)	Week 4 (*n* = 97) *M* (*SD*)	Week 7 (*n* = 95) *M* (*SD*)
All Usability Items	4.26 (0.62)*	4.32 (0.62)*	4.26 (0.67)*
Enjoyable	4.25 (0.89)*	4.43 (0.76)*	4.29 (0.85)*
Would recommend to others	4.33 (0.90)*	4.48 (0.78)*	4.46 (0.82)*
Easy to use	4.55 (0.71)*	4.53 (0.82)*	4.43 (0.72)*
Quick and easy to learn every day	4.34 (0.87)*	4.37 (0.82)*	4.26 (0.98)*
Easy to navigate	4.42 (0.85)*	4.46 (0.79)*	4.42 (0.75)*
Easy to understand	4.69 (0.60)*	4.55 (0.74)*	4.49 (0.77)*
Helps me remember where I left off	4.54 (0.80)*	4.55 (0.84)*	4.51 (0.93)*
Fits into my daily schedule	3.98 (1.04)*	3.97 (1.01)*	4.02 (1.11)*
I am making progress	3.76 (1.10)*	3.92 (0.97)*	3.83 (1.11)*
Feel helpful	4.14 (0.96)*	4.25 (0.89)*	4.25 (0.89)*
Modules can help me	4.37 (0.84)*	4.38 (0.82)*	4.32 (0.87)*
I’m learning about my ADHD	4.38 (0.96)*	4.55 (0.74)*	4.41 (0.84)*
More likely to use Inflow than see a therapist	3.59 (1.34)*	3.78 (1.39)*	3.72 (1.43)*

*p < .001 for a one sample *t* test evaluating difference from 3 (Neither Agree nor Disagree)

### Feasibility

Feasibility data from the 178 enrolled participants for whom it was available appears in Table 3 (See previous Description of Inflow App for descriptions of each activity.) During the 7 weeks of app usage, the median user opened the app 27 times, finished 1 module, completed 1 challenge, and played 12 exercises (materials with strategies to manage ADHD symptoms). The median participant’s median app session duration was 3.40 minutes. The median active use rate of Inflow is 3.43, meaning that the median participant actively interacted on Inflow with materials, challenges, or other people in the community 3.43 times per week. Despite these indicators of central tendency, as is apparent from Table 3, user engagement with the app varied significantly from person to person and the usage variables are quite skewed. Therefore, when calculating correlations involving usage data, we report these with and without outliers.

**Table 3:** App Usage Data Across 7 Weeks.

Event	*M*	*Median*	*SD*	*Min*	*Max*	*n*
No. of app sessions (total over 7 weeks)	48.07	27	70.09	3	715	178
Median app session duration (minutes)	4.0157	3.40	3.09	0.02	17.00	173
No. of exercises played (total over 7 weeks)	21.27	12	24.52	0	148	178
No. of days challenges tracked (total over 7 weeks)	22.62	4	58.09	0	529	178
No. of challenges completed (total over 7 weeks)	3.53	1	8.69	0	81	178
Community posts (total over 7 weeks)	3.71	0	9.99	0	78	178
Viewing community posts (total over 7 weeks)	9.57	3.50	17.36	0	154	178
No. of journal entries (total over 7 weeks)	7.01	3	9.48	0	72	178
Live events attended and recorded events viewed (total over 7 weeks)	1.50	0	4.51	0	50	178
Modules started (total over 7 weeks)	3.29	2	3.04	0	15	178
Modules completed (total over 7 weeks)	2.10	1	2.92	0	14	178
Active use rate	8.02	3.43	13.63	0.00	104.86	178

### Exploratory analysis: Efficacy

We first calculated change scores for each participant by subtracting pre-test scores from post-test scores such that improvement in self-reported ADHD symptoms and impairment is indicated by a negative score. The majority of participants reported less severe symptoms at post vs. baseline for inattentive (73.1%), hyperactive-impulsive (63.4%) and total ADHD symptoms (72.0%) with 9.7%, 10.8%, and 4.3% of participants reporting precisely no change for each of these symptom types, respectively. A majority also reported lower levels of impairment (66.7%) at post than at baseline with only 1.1% reporting no change.

Among the 93 users with complete and matchable efficacy data at Week 7, the average score for self-reported ADHD symptoms and impairment decreased significantly during the seven weeks of using Inflow (Table 4). We calculated effect sizes (*d*_*b*_) using the *SD* of the baseline score as the standardizer [35]. Effects on overall self-reported ADHD symptoms were in the medium range (*d*_*b*_ = -0.71 using *SD* of baseline scores). As observed in other studies of cognitive behavioral therapy [13], there was a larger change on self-reported Inattentive ADHD symptoms (*d*_*b*_ = -1.0) than on self-reported Hyperactive-Impulsive ADHD symptoms (*d*_*b*_ = -.41). Finally, we observed a small (*d* = -.46) but statistically significant decrease in self-reported impairment in major life areas during Inflow use.

**Table 4:** Comparison of Baseline and Week 7 Symptom and Impairment Scores.

	Baseline *M* (*SD*)	Week 7 *M* (*SD*)	*r*_*Baseline*, *Week 7*_	*t*_paired_ *df* = 92	*d*_*b*_ *M*_*Week7*_*-M*_*Baseline*_ */ SD*_*Baseline*_
Inattentive Symptoms (BAARS)	3.22 (0.39)	2.83 (0.59)	.41	6.71, *p* < .001	-1.0
Hyperactive-Impulsive Symptoms (BAARS)	2.60 (0.66)	2.33 (0.73)	.70	4.75, *p* < .001	-0.41
Total ADHD Symptoms (BAARS)	2.91 (0.46)	2.58 (0.59)	.58	6.39, *p* < .001	-0.71
Functional Impairment (BFIS)	5.43 (1.30)	4.83 (1.64)	.57	4.15, *p* < .001	-0.46

Note: *n* = 93; BAARS = mean Barkley Adult ADHD Rating Scale; BFIS = mean Barkley Functional Impairment Scale (15 items). All measures are self-reported. Effect size (*d*_*b*_) was calculated using the *SD* of the baseline score as the standardizer [35].

We also analyzed results from only those participants who reported a previous diagnosis of ADHD (*n* = 81). Results remained significant and were stronger within this group than within the entire sample (Inattentive *d*_*b*_ = -1.10; Hyperactive-Impulsive *d*_*b*_ = -0.47; Total ADHD *d*_*b*_ = -0.81; Impairment *d*_*b*_ = -0.63). Among the 12 participants who had not been previously diagnosed with ADHD (but believed they had the disorder), none of the changes over time were significant and effect sizes were substantially smaller (Inattentive *d*_*b*_ = -0.42; Hyperactive-Impulsive *d*_*b*_ = -0.02; Total ADHD *d*_*b*_ = -0.22) and, in one case, opposite to the expected direction (Impairment *d*_*b*_ = 0.32). Thus, changes during Inflow use appeared to be more favorable for people with a previous diagnosis of ADHD.

### Exploratory analysis: Relationship between app use and efficacy

We calculated correlation coefficients between symptom and impairment change scores and feasibility measures (app usage data) among participants for whom both sets of data were available (*n* = 87). Because several app usage variables were significantly skewed, we also calculated and reported these correlation coefficients after removing extreme outliers for each usage variable.

Correlations between the feasibility measures and the change in self-reported ADHD symptoms and functioning suggest, in general, that more *active* use of Inflow was associated with greater improvement in self-reported symptoms and impairment (Table 5). Using all available data (left portion of Table 5), a higher active use rate was significantly correlated with improvement in hyperactive-impulsive and total ADHD symptom scores. Number of exercises played, journal entries, and modules started and completed were significantly correlated with improvement in self-reported hyperactive-impulsive symptoms, total ADHD symptoms, and impairment. Viewing community posts was significantly correlated with improvement in self-reported total symptoms and impairment. Number of challenges completed was also significantly correlated with improvement in self-reported hyperactive-impulsive symptoms. Notably, the number of app sessions was not significantly correlated with changes in symptoms or impairment; and *longer* median duration of app sessions was associated with *less* positive change in self-reported ADHD symptoms. Instead, engagement with and progress through specific elements of the app were associated with more positive changes. Importantly, however, many of these significant associations were no longer significant when usage data outliers were removed (right portion of Table 5), although the direction of effects, in most cases, remained.

**Table 5:** Correlations (r) between app use rates and symptom and impairment change scores.

	Full Sample					Sample with Usage Outliers Removed				
	*n*	Inatt Change	Hyp-Imp Change	BAARS Change	BFIS Change	*n*	Inatt Change	Hyp-Imp Change	BAARS Change	BFIS Change
No. of app sessions (total over 7 weeks)	87	.01	-.13	-.07	-.10	85	-.10	-.14	-.13	-.09
Median app session duration (min)	83	.25*	.19	.25*	.17	82	.20	.19	.22*	.16
No. of exercises played (total over 7 weeks)	87	-.19	-.26*	-.25*	-.29**	86	-.12	-.18	-.17	-.21
No. of days challenges tracked (total over 7 weeks)	87	-.13	-.20	-.19	-.16	83	-.14	-.18	-.17	-.16
No. of challenges completed (total over 7 weeks)	87	-.15	-.21*	-.20	-.16	84	-.17	-.14	-.17	-.19
Community posts (total over 7 weeks)	87	-.15	-.16	-.18	-.16	81	-.17	-.04	-.12	-.07
Viewing community posts (total over 7 weeks)	87	-.18	-.21	-.22*	-.23*	85	-.10	-.06	-.09	-.12
No. of journal entries (total over 7 weeks)	87	-.20	-.30**	-.28**	-.25*	86	-.11	-.21	-.18	-.13
Live events attended and recorded events viewed (total over 7 weeks)	87	-.07	-.01	-.04	-.06	81	-.04	-.04	-.04	.04
Modules started (total over 7 weeks)	87	-.20	-.27*	-.26*	-.26*	---	---	---	---	---
Modules completed (total over 7 weeks)	87	-.19	-.28**	-.27*	-.25*	---	---	---	---	---
Active Use Rate	87	-.16	-.23*	-.22*	-.21	85	-.11	-.15	-.15	-.16

*Note*. Inatt is inattentive symptoms. Hyp-Imp is hyperactive impulsive symptoms. BAARS is the Barkley Adult ADHD Rating Scale. BFIS is the Barkley Functioning Impairment Rating Scale.

**. Correlation is significant at the 0.01 level (2-tailed).

*Correlation is significant at the 0.05 level (2-tailed).

## Discussion

In this open feasibility study, Inflow demonstrated preliminary usability and feasibility among users with a self-identified need for a CBT-based app for adult ADHD. At all three time-points (2, 4, and after 7 weeks), users who remained in the study agreed that Inflow was user-friendly, helpful, and that they would recommend it to others. The median number of app sessions per week across users was 3.86 with a median duration of 3.40. The active use rate of Inflow was 3.43, meaning that the median participant actively interacted with Inflow materials, challenges, or other people in the community about 3–4 times per week. This rate of active use compares favorably with the active use rate of 2.3 obtained in the study of PRIME, the mHealth app for young people with schizophrenia [24] and is consistent with the favorable usability ratings. Participants who provided self-reported symptom and impairment data after 7 weeks of use, on average, experienced decreases in self-reported ADHD symptoms and functional impairment. Active engagement with Inflow components—not simply frequency and duration of app use—was associated with greater improvement in self-reported symptoms and impairment. A more rigorous RCT is clearly needed to further evaluate whether use of Inflow is associated with positive change above and beyond regression to the mean or non-specific factors and to further evaluate which components are most strongly associated with any treatment-related change.

Our study joins the work of Jang and colleagues [28] in suggesting that mobile apps may be a feasible, user-friendly way of delivering psychoeducation and CBT skills to people with attention difficulties in daily life. Compared to participants who used their chatbot app over four weeks, we observed similar pre-to-post effect sizes for self-reported symptoms during our 7-week open trial. However, additional work is needed to establish the efficacy of CBT-based apps for adult ADHD.

This study provided several pieces of important data toward the design of a follow-up RCT. We now have an estimate of the standard deviation and pre-to-post effect sizes of two key potential outcomes measures, the *BAARS-IV* and *BFIS*, to inform power analysis and sample size calculation for a future study. Findings from the current study will also aid in the selection of primary outcome measures in a future trial. While we looked to past work for our usability questionnaire [31], the scale we used was not validated and, in future work, we will plan to use a validated measure such as the *mHealth App Usability Questionnaire* [37]. Finally, we clearly need a more effective procedure to match participant survey data across time-points. We selected the unique identifier method to enhance confidentiality, but in the future we may use a consistent personal identifier such as email address to reduce the likelihood of mismatches.

Reflecting on the current study also raised several additional issues that must be addressed in the design of a future RCT and these observations may also be useful to other researchers developing and testing mHealth apps designed to bring CBT into the daily lives of people with mental health disorders. Such studies present methodological challenges and require close collaboration between researchers from different disciplinary perspectives [38]. First, we must carefully consider the inclusion criteria for the study. In the current study, we did not require a self-reported ADHD diagnosis and yet we found that significant changes in symptoms and impairment during app use only occurred for the previously diagnosed group—a group with substantial self-reported symptoms and impairment. Although there are clear advantages to testing a very rigorously diagnosed clinical sample—using, for example, structured diagnostic interviews—such procedures might limit both the sample size and the generalizability of the study results, especially given the constraints of online recruitment. A recent RCT of Zemedy, the CBT-based app for irritable bowel syndrome, provides a potentially useful model in which the researchers used the results from multiple self-report scales to create a multi-part screening protocol [25]. Participant comorbidity is also important to consider in selecting inclusion criteria. Furthermore, the sample in the current study was majority White and more highly educated than the general U.S. population. A next-step study should recruit sufficient numbers of people with diverse racial and ethnic identities as well as people with more diverse education levels in order to adequately evaluate the efficacy of Inflow in these groups.

Second, we must carefully consider which additional constructs should be assessed as primary or secondary outcome measures. In addition, the RCT should include a specific measure of potential harms or side effects of the intervention, given that CBT can be associated with adverse effects [18]. Finally, we will need to decide on the most appropriate comparison or control group for a future RCT and will need to employ more rigorous data analytic methods with reduced potential for bias, such as multiple imputation to handle missing data from drop outs. Importantly, multiple imputation will rely on the inclusion of additional baseline measures to use in imputing the missing data.

As a feasibility study, the current work has a number of limitations, some of which are outlined in the preceding discussion of issues to address in a future RCT. One limitation not yet discussed is the dropout rates associated with mHealth app interventions. Of participants who consented, about 54% could be considered drop outs before the Week 7 assessment: 14% failed to successfully enroll with the Inflow app, 36% dropped out before Week 2, 3% between Week 2 and 4, and only 1% dropped out between Week 4 and 7. We anticipated a sizable dropout rate, despite our use of a potentially motivated recruitment pool, and took it into account when designing our recruitment plan and target sample size. Indeed, our dropout rate is not unexpected given the typically low retention rates for app use [39,40]. For example, in a recent survey, the global app retention rate after 30 days across all categories was 4.2% [40]. In past studies of app-based interventions for chronic disease, the average dropout rate was 43% [41]. Higher rates of dropout might also be related to the fully online recruitment procedures used in the current study (47% on average for fully online trials reported by Mathieu et al. [42])—another factor to consider in designing the subsequent RCT.

Although not uncommon, high dropout rates are problematic for drawing conclusions from research. Drop out can be an indicator of a lack of feasibility of the intervention. Although we did not specify dropout rate, *a priori*, as a measure of feasibility, some dropouts may have occurred because participants did not find the app user-friendly and helpful, thereby positively biasing our usability and other findings. As such, it is important to emphasize that the findings are only representative of the participants who stayed in the study. We were encouraged to observe that dropout did *not* appear to be associated with baseline symptom severity, impairment, or comorbidity, and so we have no evidence that the app is less accessible to those participants most in need of it.

Finally, high dropout may positively bias estimates of efficacy in a future randomized controlled trial. In addition to preventing dropout through the design of a future study and taking it into account in statistical analyses, future development of Inflow and other CBT-based apps will need to focus on continuing to boost participant engagement early in the user lifecycle [43]. And, while dropout during clinical trials is not a new problem, it is one that certainly reflects the “real world” of both traditional therapy and mHealth interventions [44]. Importantly, even when dropout rates are less favorable for app-based interventions than for traditional therapy, the accessibility of apps relative to traditional therapy could nonetheless result in delivering treatment to greater numbers of people in need.

The current study reports the preliminary usability and feasibility of the Inflow app and paves the way for an RCT to better evaluate its effects on symptoms and impairment. We hope that this mHealth tool can increase access to the benefits of CBT for people with ADHD around the world.
